# Salvage chemoradiation therapy for recurrence after radical surgery or palliative surgery in esophageal cancer patients: a prospective, multicenter clinical trial protocol

**DOI:** 10.1186/s12885-020-07315-0

**Published:** 2020-09-14

**Authors:** Xiao Chang, Lei Deng, Wenjie Ni, Chen Li, Weiming Han, Lin-rui Gao, Shijia Wang, Zongmei Zhou, Dongfu Chen, Qinfu Feng, Jun Liang, Nan Bi, Jima Lv, Shugeng Gao, Yousheng Mao, Qi Xue, Zefen Xiao

**Affiliations:** 1 Department of Radiation Oncology, National Cancer Center/National Clinical Research Center for Cancer/Cancer Hospital, Chinese Academy of Medical Sciences and Peking Union Medical College, No. 17 South Panjiayuan lane, Chaoyang District, Beijing, 100021 China; 2 Department of Thoracic Surgery, National Cancer Center/National Clinical Research Center for Cancer/Cancer Hospital, Chinese Academy of Medical Sciences and Peking Union Medical College, Beijing, China

**Keywords:** Esophageal neoplasm, Locoregional recurrence, R1/R2 resection, Chemoradiation therapy, Palliative management

## Abstract

**Background:**

Currently, adjuvant therapy is not recommended for patients with thoracic esophageal squamous cell cancer (TESCC) after radical surgery, and a proportion of these patients go on to develop locoregional recurrence (LRR) within 2 years. Besides, there is no evidence for salvage chemoradiation therapy (CRT) in patients with residual tumor after esophagectomy (R1/R2 resection). In addition, factors like different failure patterns and relationship with normal organs influence the decision for salvage strategy. Here, we aimed to design a modularized salvage CRT strategy for patients without a chance of salvage surgery according to different failure patterns (including R1/R2 resection), and further evaluated its efficacy and safety.

**Methods:**

Our study was designed as a one arm, multicenter, prospective clinical trial. All enrolled patients were stratified in a stepwise manner based on the nature of surgery (R0 or R1/2), recurrent lesion diameter, involved regions, and time-to-recurrence, and were further assigned to undergo either elective nodal irradiation or involved field irradiation. Then, radiation technique and dose prescription were modified according to the distance from the recurrent lesion to the thoracic stomach or intestine. Ultimately, four treatment plans were established.

**Discussion:**

This prospective study provided high-level evidence for clinical salvage management in patients with TESCC who developed LRR after radical surgery or those who underwent R1/R2 resection.

**Trial registration:**

Prospectively Registered. ClinicalTrials.govNCT03731442, Registered November 6, 2018.

## Background

According to the 2019 National Comprehensive Cancer Network (NCCN) guidelines for esophageal cancer [1], adjuvant treatment is not recommended for patients with thoracic esophageal squamous cell cancer (TESCC) who received radical surgery as their first treatment, regardless of the T and N status. However, the recurrence rate is as high as 23.8–58%, and the median time to recurrence is about 10.5 months [2–6]. Even in Japan where three-field lymphadenectomy is the preferred treatment option, 24–46% patients go on to experience recurrence after R0 resection, which is the main cause of surgical treatment failure [7–10]. Besides, in patients with residual tumor (R1/R2), salvage chemoradiation therapy (CRT) is recommended as the main component of palliative management for locoregional recurrence (LRR) disease. However, data of large samples or high-level evidence are still lacking.

Our previous retrospective analysis [11] indicated that most patients developed lymph node recurrence in the supraclavicular (25.8%) and upper mediastinal (44.4%) regions, and those who underwent salvage CRT had significantly better survival than those that underwent radiotherapy alone, chemotherapy, or best supportive care. Similar results were found in other studies [12–15]. Overall survival (OS) directly depended on failure patterns and corresponding treatment strategies, so prospective clinical trials were necessary for screening of specific patients to attain survival benefit from the optimal salvage strategy.

This study was aimed to design a modularized salvage CRT strategy for patients unsuited for salvage surgery based on different failure patterns (including R1/R2 resection) and further evaluate its efficacy and safety.

## Methods/design

### Study design and objectives

**Fig. 1:**
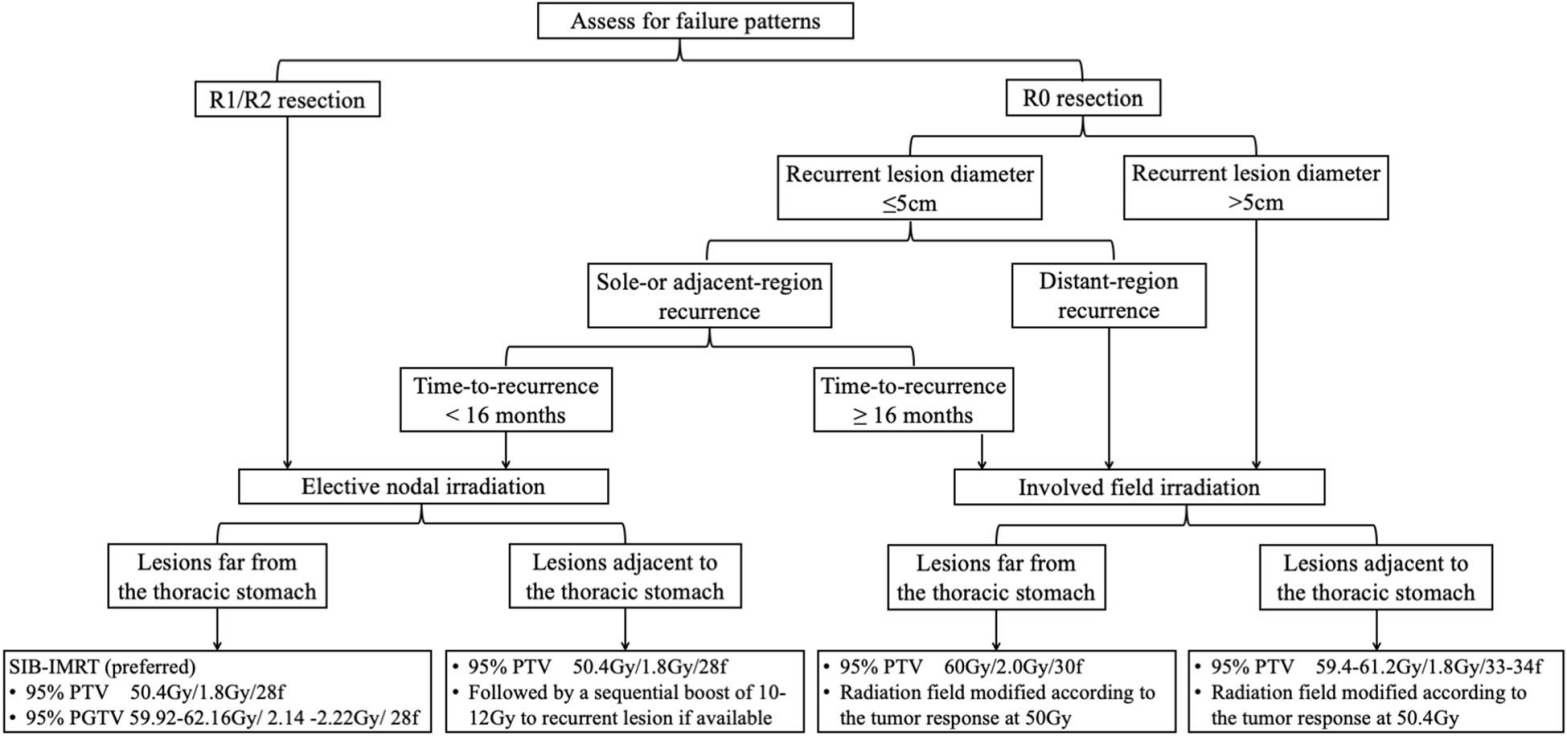
Flow chart of the trial

The current study was designed as a one-arm, multicenter, prospective clinical trial. The enrolled patients were stratified in a stepwise manner based on the nature of surgery (R0 or R1/2), recurrent lesion diameter, involved regions, and time-to-recurrence, and were further assigned to undergo either elective nodal irradiation (ENI) or involved field irradiation (IFI). Then, radiation technique and dose prescription were modified according to the distance from the recurrent lesion to thoracic stomach or intestine. Ultimately, four treatment plans were established. A flow chart of the study overview is shown in Fig. 1.

The primary end point is the 1-, 2-, and 3-year OS. The secondary end points include the 1-, 2-, and 3-year rates of progression-free survival (PFS), completion rates, out-field recurrence, and toxicity profiles.

The study began on November 2018, and patients will continue to be included until November 2022.

### Patient population

Patients enrolled thus far mainly comprise untreated patients after LRR or palliative surgery. The inclusion criteria include: (1) R1/R2 resection, (2) LRR after radical surgery, (3) out-field LRR after adjuvant chemoradiation or radiotherapy, (4) LRR after adjuvant chemotherapy, (5) no prior therapy after LRR, (6) age 16–70 years, (7) good general condition (i.e., Karnofsky Performance Status [KPS] ≥70)], (8) normal complete blood count (CBC), especially white blood cell count > 4.0*10^9/L, (9) satisfactory liver and kidney functions.

The exclusion criteria include: (1) prior malignancies within 5 years, (2) pregnant status or lactation, (3) history of drug allergy, (4) refused informed consent (5) non-regional lymph node (except for metastasis to supraclavicular or celiac lymph nodes) or distant metastasis (including metastasis to organs including bone, lung or liver etc.) (6) severe cardiovascular diseases, infections, active ulcerations, diabetes mellitus with unstable blood sugar, and mental disorders.

### Recurrence

Tumor residue includes positive pathological margins of the specimens (R1) and incomplete tumor resection during the operation (R2). LRR is defined as recurrence at sites of the anastomosis, tumor bed, mediastinal lymph nodes, or para-gastric lymph nodes (including nodes adjacent to the cardia or along the course of the left gastric artery). Recurrence in the deep cervical, supraclavicular, or celiac regions are also defined as regional relapse. Distant metastasis was defined as metastasis in the liver, lung, bone, and pleura; subcutaneous metastasis; and other nonregional lymph node metastasis such as axillary and inguinal lymph nodes. If a second recurrence was detected within 4 weeks after the first occurrence, it was considered synchronous. Once suspicious recurrent lesions are identified on imaging, biopsy is attempted. The diagnostic standard for imaging should meet the criteria of significant enlargement or increase in the number of existing lymph nodes, or the appearance of the new lymph nodes compared with previous examinations. Otherwise, positron emission tomography-computed tomography (PET-CT) clearly diagnoses recurrence through metabolic activity and imaging features.

**Fig. 2:**
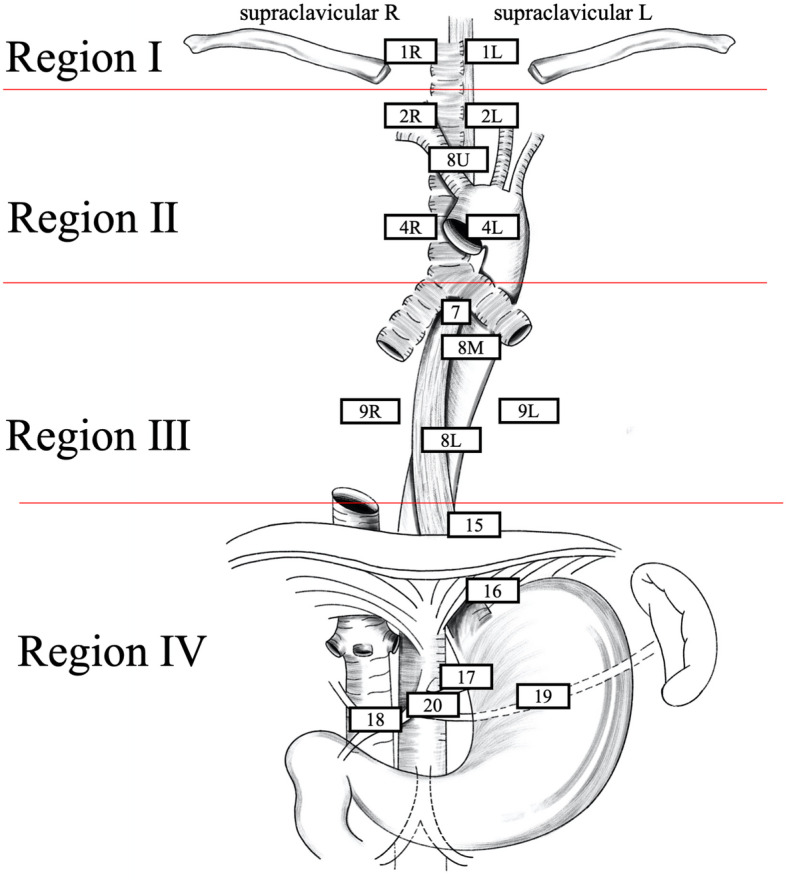
Illustration depicting reclassified regions

To comprehensively describe the design of target volume, the 8th American Joint Committee on Cancer (AJCC) regional lymph node stations [16] were reclassified into four regions (Fig. 2). Region I includes the area above the sternal notch, including the supraclavicular space and No. 1 lymphatic drainage region; region II includes the mediastinal No. 2, 4, and 8 U lymphatic drainage regions; region III includes mediastinal No. 7, 8 M/Lo, and 9 lymphatic drainage regions; and region IV includes the abdominal No. 15–20 lymphatic drainage regions. Close region recurrence was defined as recurrences within the sites of (1) regions I and II, (2) regions II and III, (3) regions III and IV, or (4) regions I and III. Distant regional metastasis was defined as recurrences at the both sites of regions I and IV or region II and IV.

### Radiotherapy

The planning CT was recommended to be fused with planning magnetic resonance imaging (MRI) or PET-CT, if available, to further improve the contouring accuracy. The gross tumor volume (GTV-T) or metastatic regional nodes (GTV-N) is defined as the residual tumor, tumor-bed recurrence, or metastatic lymph node. The planning gross tumor volume (PGTV) is created by expanding GTV-T or GTV-N with a uniform 0.5-cm margin. As for delineation of clinical target volume (CTV), both IFI and ENI were adopted.

**Fig. 3:**
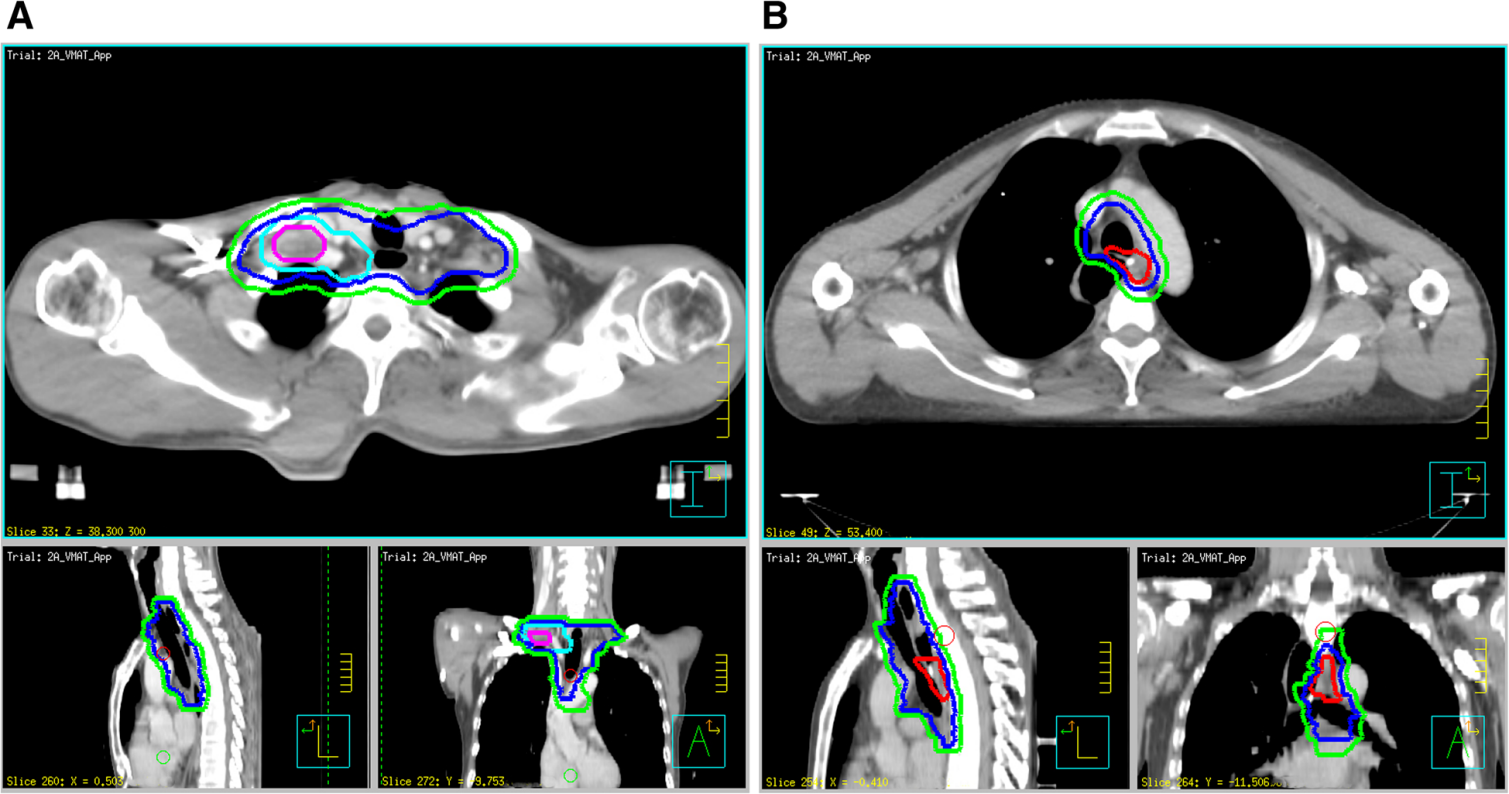
Examples of target volumes delineated in elective nodal irradiation (ENI) pattern are shown based on the planning computed tomography (CT) scans. **a** Lesions far from the thoracic stomach recurred in right supraclavicular space. The ENI field includes bilateral supraclavicular space, 1R/L, 2R/L, 4R/L, partial 7, and 8 U/M. **b** Lesions adjacent to the thoracic stomach recurred in Station 8 U, ENI field includes bilateral supraclavicular space, 1R/L, 2R/L, 4R/L, partial 7, and 8 U/M. Pink outlines GTV-N, Red outlines GTV-T, blue outlines CTV, sky blue outlines PGTV, and green outlines PTV

In the ENI group, the principle to design prophylactic target volume of high-risk lymphatic drainage regions basically comprised GTV-T/GTV-N plus a 3.0–5.0-cm craniocaudal and 0.6-cm horizontal margin. For recurrence in regions I or II, CTV comprised the region with the upper boundary at the upper margin of the T1 vertebral body or 1.0–1.5-cm superior to GTV-N and lower boundary in the 2.0–3.0-cm inferior to the carina, including the supraclavicular space and No. 1, 2, 4, 7, and 8 U stations. For recurrence in region III, the CTV comprised the region with upper boundary at the level of the clavicular head and lower boundary in the margin 2.0-cm inferior to the carina or 1.0–1.5-cm inferior to GTV-N, including No. 2, 4, 7, and 8 U/M stations. For recurrence in region IV, CTV comprised the region with upper boundary in the 1.0–1.5-cm superior to GTV-N and lower boundary in the celiac axis or 1.5-cm inferior to GTV-N, including No. 15–20 stations. The technique of intensity-modulated radiation therapy (IMRT) with simultaneously integrated boost (SIB) or sequential boost was modified according to the safety of the thoracic stomach or intestine. Figure 3a shows the SIB-IMRT being applied to a recurrent lesion far from the thoracic stomach with a prescription dose of PTV 50.4 Gy/1.8 Gy/28 f and PGTV 59.92–62.16 Gy/2.14–2.22 Gy/28 f. Figure 3b shows the IMRT with sequential boost applied to a recurrent lesion close to the thoracic stomach with a prescription dose of PTV 50.4 Gy/1.8 Gy/28 f and a sequential boost to PGTV 10–12 Gy/1.8–2 Gy/5–7 f.

**Fig. 4:**
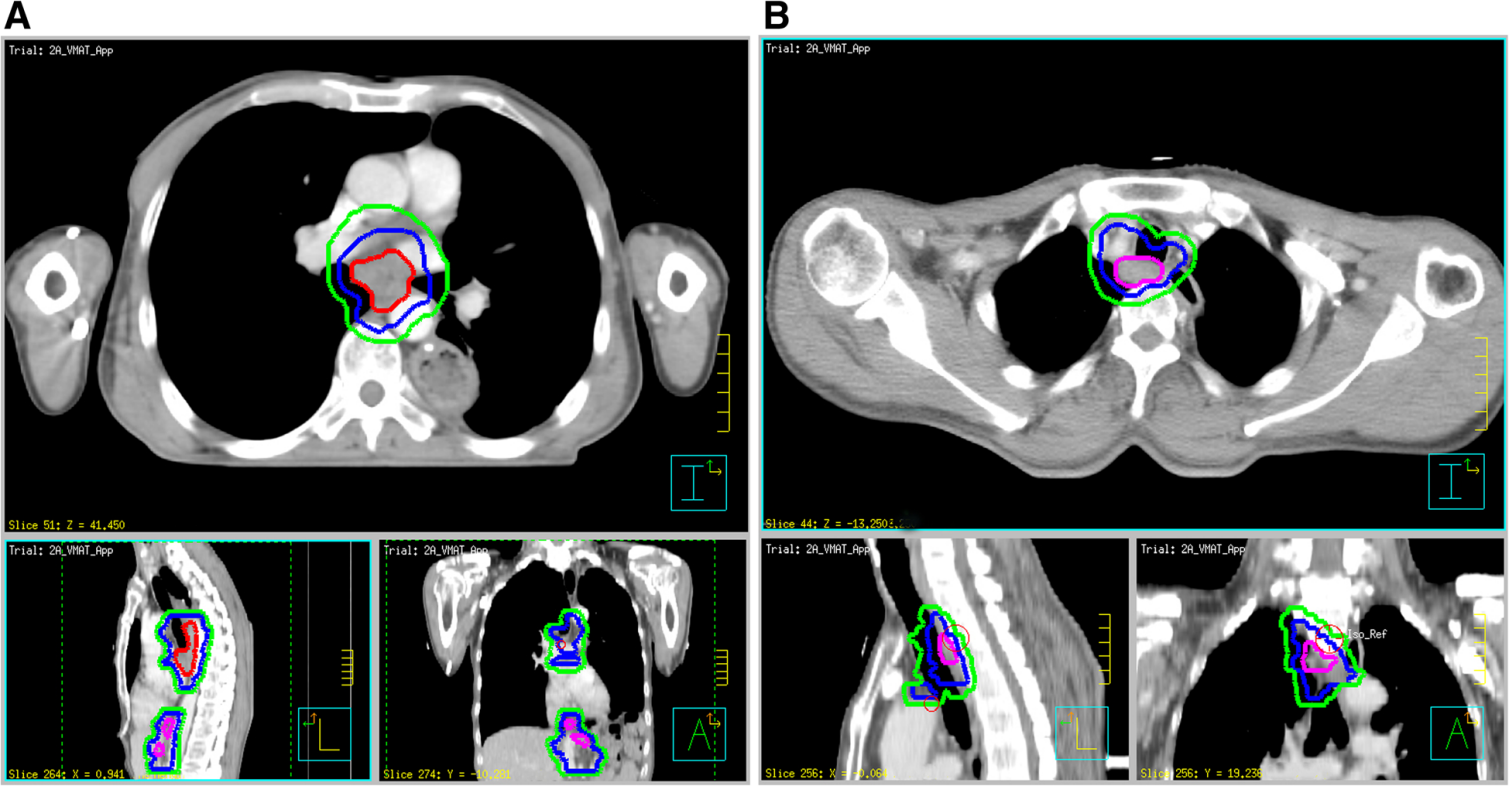
Examples of target volumes delineated in involved field irradiation (IFI) pattern are shown based on the planning computed tomography (CT) scans. **a** Lesions which were > 5 cm and far from the thoracic stomach recurred in Station 7, 8 M/L and 15–17. The IFI field includes Station partial 4R/L, 7, 8 U/M, and 15–17. **b** Lesions adjacent to the thoracic stomach recurred in Station 2R and palliative CRT was performed because of its time-to-recurrence > 16 months. The IFI field only includes peritumor regions. Pink outlines GTV-N, Red outlines GTV-T, blue outlines CTV, and green outlines PTV

In the IFI group, CTV only consisted of GTV-T/GTV-N plus a 0.6–0.8-cm horizontal margin and 1.0–1.5-cm craniocaudal margin. No prophylactic irradiation was delivered to any lymph node drainage regions. For lesions located away from the thoracic stomach (Fig. 4a), the prescribed dose was 60 Gy/2 Gy/30 f, and for lesions close to the thoracic stomach (Fig. 4b), the prescribed dose was 59.4–61.2 Gy/1.8 Gy/33–34 f. Planned chest CT at 50 Gy followed by the radiation field should be modified according to the tumor response. The PTV is derived from CTV with a uniform 0.5-cm margin.

Bilateral lungs, spinal cord, heart and reconstructed thoracic stomach are identified as organs at risk (OAR) and should be delineated on planning CT. The dose to the organs at risk of IMRT technique was controlled as follows: maximal dose to the spinal cord was < 45 Gy, the percentage of irradiated bilateral lung volume exceeding 20 Gy was ≤28%, the mean dose of irradiated bilateral lung was ≤17 Gy, the percentage of irradiated heart volume exceeding 40 Gy was < 30%, the percentage of irradiated stomach volume exceeding 40 Gy was ≤40%, and no hotspot present on the gastric tube. If the V50 thoracic stomach> 50%, the total dose should be reduced up to 45 Gy/1.8 Gy/25 f.

### Chemotherapy

Concurrent with radiotherapy, patients will receive two cycles of paclitaxel (135–150 mg/m^2^, d1) and lobaplatin (30 mg/m^2^, d1–2, total dose should not exceed 50 mg) or nedaplatin (50 mg/m^2^, d1–2) every 3 weeks. Injection with polyethylene glycol recombinant human granulocyte colony-stimulating factor (PEG-rhG-CSF) for prophylaxis will be recommended 48 h after chemotherapy. Two or four cycles of consolidated chemotherapy are recommended within 3 months for patients meeting the following criteria:(1) KPS ≥ 70, (2) able to swallow more than semisolid food or with nasal feeding, and (3) weight loss ≤5% in 1–2 months after radiotherapy. The regimen is the same as that of the concurrent phase. However, if patients have previously received taxane- and platinum-based chemotherapy regimens and go on to develop LRR within 6 months, the second-line regimen of chemoradiotherapy is recommended.

### Toxicity and adverse events

The Radiation Therapy Oncology Group (RTOG) and Common Terminology Criteria of Adverse Events (CTCAE) version 4.0 was applied for grading all treatment-related toxicities, which should be recorded in the electronic patients’ case report form (CRF). Any serious adverse event (SAE) should be dealt with immediately and reported to our institutional ethical review committee within 24 h.

The second cycle of chemotherapy should be adjusted based on the most severe chemotherapy-related toxicity after the first cycle, and it should only be administered when the toxicity subsides to baseline or grade 0/1. The regimen should be administered at 80% of the original dose in case of grade 3 hematogenous toxicity, and chemotherapy should be terminated if grade 4 hematogenous toxicity, grade 2 hepatic or renal dysfunction, and other non-homological grade 3 toxicities occurred. If grade 3/4 radiation pneumonitis develops, both radiotherapy and concurrent chemotherapy should be terminated.

### Follow-up

Tumor regression should be evaluated according to the Response Evaluation Criteria in Solid Tumors (RECIST) Version 1.1 within 1–2 months, and response to treatment should be documented. A 5 years followed-up after treatment should be completed in all patients, with the time interval of every 3 months in the first 2 years, every 6 months in 3–5 years, and once a year thereafter.

Routine items of follow-up should include the following: (i) Any symptom related to the disease or treatment, such as cough, fever, hoarseness or dysphagia. (ii) Blood test, including CBC and comprehensive blood chemistry profile if necessary. (iii) Imaging examinations, including the contrast-enhanced CT; ultrasound of the neck and abdomen; upper gastrointestinal contrast. (iv) Disease progression and its subsequent treatment, survival status nutrition, life quality, and late toxic effects should also be documented.

### Statistical analysis & sample size considerations

A chi-square test was used to compare categorical data, with or without correction for continuity. Salvage OS was defined as the date of receiving salvage CRT to the date of death or the last clinical follow-up. DFS was measured from the date of receiving salvage CRT to the date of progression or death from any cause or last clinical follow-up. Cumulative survival rates were estimated using the Kaplan–Meier survival curve and compared using the log-rank test.

This trial is designed as a one-arm prospective study, using PASS 11.0.7 to estimate the sample size. We assume that the estimated 1-year OS of the ENI arm and IFI arm will be 70 and 60%, respectively. Assuming a two-sided significance level of 0.05 and a power of 0.80, the sample size of the ENI group and IFI group are estimated to be 83 and 199, respectively. Therefore, a total of 282–300 patients would be needed considering patient loss in each group.

### Ethics

The study protocol has been approved by the ethics committee of the Chinese Academy of Medical Sciences (18–175/1753). The study has been registered in ClinicalTrails.gov (NCT03731442).

## Discussion

Although the 2016–2019 NCCN guidelines recommended CRT as the optimal treatment for patients who developed LRR with prior esophagectomy or received R1/R2 resection, the principle of target volume delineation or dose prescription for salvage CRT are still unclear. In addition, radiation oncologists may find it difficult to screen patients with potential curative possibilities according to failure patterns and recurrence time to attain better patient survival. Therefore, more prospective clinical trials are necessary.

Ni et al. [11] reported that postoperative pTNM stage and salvage treatment regimen were independent prognostic factors for LRR in esophageal cancer after surgery. Besides, close follow-up after surgery for early detection and timely treatment were also crucial factors. However, the optimal radiation dose for recurrent esophageal cancer has not yet been determined. Nemoto et al. [17] reported that the median survival period of patients who received a radiation dose of ≤60 Gy was 2 months longer than those who received > 60 Gy, although this difference was not statistically significant. However, Zhang et al. [18] reported that TESCC patients with LRR showed better OS and PFS in the group of ≥60 Gy than those in the group of < 60 Gy, and Shioyama et al. [19] also demonstrated that the high-dose group (> 50 Gy) was associated with better survival. Our previous data [11] showed that patients receiving > 60 Gy irradiation dose had a significantly greater 5-year OS than those who received ≤60 Gy (25.3% vs. 13.8%, *P* = 0.026). Three-dimensional conformal radiotherapy or IMRT could significantly reduce these toxicities because of better dose distribution between the tumor and normal tissue [20–23], and the SIB technique allowed an escalation dose specifically toward the tumor without over irradiating the OARs [23, 24]. Our prospective phase I/II trial [25] supported the safety and efficacy of the dose patterns adopted in this trial (95% PGTV/PTV 59.92 Gy/ 50.40 Gy/28 f, EQD2 = 60.62 Gy). In addition, for patients who are intolerant to SIB-IMRT, concurrent chemoradiotherapy with a sequential boost of about 10 Gy was adopted. Welsh et al. [24] reported that 50% patients experienced local failure and 90% LRR cases were within GTV after definitive CRT with a prescription dose of 50.4 Gy. This result indicated that the local control rate was unsatisfactory and therapeutic intensification should be carried out for the primary tumor. Therefore, in order to keep the toxicity level stable, we speculated whether it was possible to improve the local control rate and prolong survival by appropriately increasing the radiotherapy dose.

Although CRT was preferred, the role of chemotherapy in palliative management remains controversial. Nemoto et al. [17] reported that combined chemotherapy was correlated with a better 2-year local control rate, but failed to show better survival. However, previously noted trial RTOG 8501 [26, 27] showed that the 5-year OS of definitive radiotherapy with or without chemotherapy was 26 and 0% (*P* < 0.001), respectively. Our findings appear consistent with other studies [11–15] and have indicated that CRT correlates with better survival than radiotherapy alone and is well tolerated in patients who developed LRR. Further, it was also unclear whether patients should receive consolidation chemotherapy. A propensity score-matched analysis [28] showed that consolidation chemotherapy did not further prolong PFS and OS following definitive CRT, and no prospective randomized clinical trials supported the addition of consolidation chemotherapy following salvage CRT. However, there was still high risk of LRR with synchronous distant metastases [3, 5, 7–10], so consolidation chemotherapy was only recommended to patients who has a good general status and responded well to the primary treatment.

However, concerning the target volumes of CRT for esophageal cancer, there is no global consensus regarding whether ENI or IFI should be performed [29–34]. In this trial, target volumes were determined by the goal of treatment. For LRR patients with potential curable possibility, prophylactic irradiation to high-risk lymph node regions should be considered because of the following reasons: (1) The median time to recurrence is short, and most studies reported 50% patients develop recurrence within 7–12 months. The median time to recurrence in our hospital was even shorter (7 months), and we rechecked cases to find that that a major proportion of patients with LRR were identified by clinical examinations and close follow-up without any symptoms such as dysphagia, obstruction, or pain. (2) The lymphatic metastasis of esophageal cancer occurred early, and lymph node dissection is known to be difficult given the complex anatomy of the upper mediastinum. (3) The recurrence rate in multiple lymphatic regions was high. Ni et al. [11] reported that > 50% patients had recurrence in multiple regions of the upper mediastinum. For patients with widespread recurrence or giant tumor bulk, IFI was mainly applied to relieve symptoms, achieve high completion rate, and thereby prolong survival.

## Data Availability

Not applicable – data collection is still ongoing.

## Abbreviations

TESCC: Thoracic esophageal squamous cell cancer
LRR: Locoregional recurrence
CRT: Chemoradiation therapy
ENI: Elective nodal irradiation
IFI: Involved field irradiation
NCCN: National comprehensive cancer network
OS: Overall survival
SIB: Simultaneously integrated boost
IMRT: Intensity-modulated radiation therapy
KPS: Karnofsky performance status
CBC: Complete blood count
PET-CT: Positron emission tomography-computed tomography
MRI: Magnetic resonance imaging
AJCC: American joint committee on cancer
GTV-T: Gross tumor volume
GTV-N: Metastatic regional nodes
PGTV: Planning gross tumor volume
CTV: Clinical target volume
PTV: Planning target volume
OAR: Organ at risk
PEG-rhG-CSF: Polyethylene glycol recombinant human granulocyte colony-stimulating factor
RTOG: Radiation therapy oncology group
CTCAE: Common terminology criteria of adverse events
CRF: Case report form
SAE: Serious adverse events
RECIST: Response evaluation criteria in solid tumors
